# Small-Area Factors and Their Impact on Low Birth Weight—Results of a Birth Cohort Study in Bielefeld, Germany

**DOI:** 10.3389/fpubh.2020.00136

**Published:** 2020-04-28

**Authors:** Lisa Wandschneider, Odile Sauzet, Jürgen Breckenkamp, Jacob Spallek, Oliver Razum

**Affiliations:** ^1^Department of Epidemiology and International Public Health, School of Public Health, Bielefeld University, Bielefeld, Germany; ^2^Center for Statistics, Bielefeld University, Bielefeld, Germany; ^3^Department of Public Health, Faculty of Social Work, Health, and Music, Brandenburg University of Technology Cottbus–Senftenberg, Senftenberg, Germany

**Keywords:** low birth weight, small-area analysis, multilevel analysis, virtual audit, noise pollution, fine particulate matter, socioeconomic deprivation, cohort study

## Abstract

**Introduction:** The location of residence is a factor possibly contributing to social inequalities and emerging evidence indicates that it already affects perinatal development. The underlying pathways remain unknown; theory-based and hypothesis-driven analyses are lacking. To address these challenges, we aim to establish to what extent small-area characteristics contribute to low birth weight (LBW), independently of individual characteristics. First, we select small-area characteristics based on a conceptual model and measure them. Then, we empirically analyse the impact of these characteristics on LBW.

**Material and methods:** Individual data were provided by the birth cohort study “Health of infants and children in Bielefeld/Germany.” The sample consists of 892 eligible women and their infants distributed over 80 statistical districts in Bielefeld. Small-area data were obtained from local noise maps, emission inventory, Google Street View and civil registries. A linear multilevel analysis with a two-level structure (individuals nested within statistical districts) was conducted.

**Results:** The effects of the selected small-area characteristics on LBW are small to non-existent, no significant effects are detected. The differences in proportion of LBW based on marginal effects are small, ranging from zero to 1.1%. Newborns from less aesthetic and subjectively perceived unsafe neighbourhoods tend to have higher proportions of LBW.

**Discussion:** We could not find evidence for negative effects of small-area factors on LBW, but our study confirms that obtaining adequate sample size, reliable measure of exposure and using available data for operationalisation of the small-area context represent the core challenges in this field of research.

## Introduction

Health inequalities on the small-area level have gained, importance over the past decades in Germany: while large-scale differences between East and West Germany are decreasing, small-area inequalities are increasing e.g. on the city district or neighbourhood level (1, 2). Thus, the location of residence represents a dimension of social inequalities that affects health related resources and stressors and in consequence individual health (3). The small-area context has an impact on mortality, morbidity and health behaviour, as consistent evidence demonstrates. Perinatal development and infant health might already be affected by the residential environment. Environmental factors can induce psychological and physiological processes (4, 5). Direct physiological processes are often linked to environmental hazards such as air and noise pollution. Psychological processes can be provoked by any adverse environmental factor such as noise and air pollution, deprivation, built environment or social resources and might trigger additional physiological processes (4). Protective environments, such as neighbourhood greenness, social contacts or high walkability, can reduce maternal stress and thus affect intrauterine development positively (5). In consequence, researchers need to look beyond the well-documented maternal risk factors to explain disparities in adverse birth outcomes like low birth weight (LBW) and preterm birth (PTB), which are valuable public health indicators for maternal and neonate health (6).

Until now, the underlying pathways of the small-area's impact on individual health and birth outcomes in particular are unknown and theoretical approaches with hypothesis-driven analyses are missing. More profound and theory-based approaches are needed to identify and understand the underlying mechanisms of inequalities (7, 8). Within the context of birth outcomes and small-area effects, systematic reviews in the US, UK and France emphasised crucial covariates that might lead to confounded results if neglected: maternal age, obstetric history, ethnicity, socio-economic status, body mass index (BMI), smoking, alcohol consumption, infections, chronic diseases, and congenital abnormalities (9–12). When considering environmental noise, air pollution needs to be adjusted for (13). Moreover, the small-area level data structure varies widely, even on the national level, and the data availability is limited. Most of the research concerning area effects on birth outcomes is realised in the US, the UK and France (14, 15) but generalisability of these finding to the German context is limited.

To address these challenges, we aim to establish to what extent small-area characteristics contribute to LBW, independent of individual characteristics. First, we select small-area characteristics on the basis of a conceptual model and assess them. Then, we empirically analyse the impact of these characteristics on LBW.

## Materials and Methods

### Study Design and Population

We perform a secondary data analysis based on the conceptual model of the small-area effects on health by Voigtländer et al. (3). The baseline survey of the birth cohort study “Health of infants and children in Bielefeld/Germany” (BaBi study) provides the individual data. The BaBi study is a population-based birth cohort with an explicit social-epidemiologic focus. It aims to examine the development of health inequalities from birth to childhood in the city of Bielefeld, Germany, with a special focus on the social, cultural and migration background. From 2013 to 2016 women were approached during pregnancy or within eight weeks after birth in all obstetric clinics in Bielefeld and via gynaecologists as well as midwives (16). Our sample includes cases that provide valid perinatal data (*n* = 71 missing cases) addresses (*n* = 6 missing cases) and only singleton births (*n* = 19 multiple births excluded), resulting in a total sample size of *n* = 892.

Individual study data is linked to small-area data, whereas the small-area context is defined by the statistical districts of the city of Bielefeld. Small-area level data sources encompass the online portal on environmental noise in North Rhine-Westphalia (NRW) (17), the online air pollutant emission inventory in NRW (EKAT) (18), Google Street View (GSV), the civil registry and employment statistics. Linking these rich data sources to a birth cohort study and thereby investigating such diverse small-area level characteristics and their impact on LBW makes the present study innovative, especially in the German context but also on an international scale as multiple small-area characteristics are usually examined individually.

### Conceptual Model

The conceptual model understands the location of residence as a determinant of social inequality affecting the individual health through resources and stressors on the small-area context. The major strengths of the model are the explicitly stated underlying pathways and that it is not restricted to a specific health outcome (19).

The fundamental hypothesis states that social inequalities (macro level) structure the resources and stressors on the small-area context (meso level) which in turn affect the individual response and internalisation (micro level) (3). The small-area level encompasses the social structure and resources/ stressors; they are interdependent and vary by the location of residence. The social structure of the small-area context is formed by the composition of the population, such as the educational level, ethnic diversity and unemployment rate. The social structure affects the available health-related resources and stressors on the small-area context which can in turn influence the social structure. Thus, the social structure and resources and stressors of the small-area context are linked by a reciprocal association. Resources and stressors are divided into four domains: physical environment (e.g., green areas or air quality), markets (rental levels or availability of foods), institutions (e.g., health care), and social capital (e.g., in form of trust, participation) (3). The small-area context can affect individual health through three different pathways. First, it defines the dose of exposure to environmental hazards that influence the individual health directly, such as noise or air pollution. Second, the resources and stressors of the small-area context can affect health indirectly by influencing individual psychosocial aspects (e.g., experienced stress or support) and individual health-related behaviour (e.g., smoking habits). The third pathway states that the small-area context has an impact on personal resources that in turn influence the psychosocial factors described above, whereby personal resources can encompass socioeconomic factors (e.g., income) as well as physical disposition (gestational age, primiparity). In consequence, the effect of resources and stressors of the small-area context can vary by living situation, personal resources and individual coping capacity (3).

The application to the explicit subject matter and available data sources required some adaptations of the conceptual model:

1. The original model conceptualises the small-area's impact on populations, we consider the mother and the newborn as one biological unit. While the outcome (LBW) is measured for the newborn, the hypothesised influencing factors affect the mother and unborn. As the birth weight is measured directly after birth, postnatal factors that only affect the newborn can be omitted.
2. The macro level will not be considered. The social, political, economic, geographic, and climatic circumstances are assumed to be equal in the study region.

Based on the conceptual model, the state of research on associated factors of (low) birth weight (9–12) and considering the availability in the data sources, we selected small-area variables and individual factors to control for. To avoid over-specification of the statistical model, we conducted a bivariate analysis using t-tests (for continuous variables) and Chi-squared tests (for categorical variable) for statistical selection and reduction of controlling variables (Supplementary Material 1).

We hypothesise that the small-area characteristics, i.e.,

- environmental hazards operationalised as daily noise due to road traffic and fine particulate exposure due to traffic,
- resources and stressors operationalised as the aesthetic of the built environment and the perceived risk of criminality during daytime,
- social structure operationalised as the deprivation index,

have an adverse effect on LBW, such as increasing the proportion of LBW, which is independent of individual level, maternal, and pregnancy-related characteristics.

**Figure 1:**
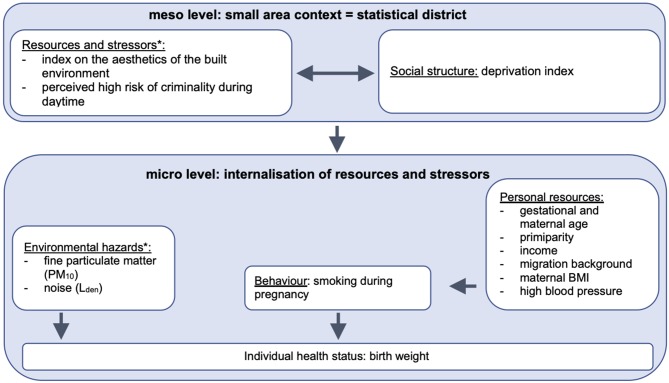
Statistical model on the impact of small-area and individual level characteristics on birth weight. *The variables describe small-area level characteristics but have been measured individually for each participant. The variables have not been aggregated to the statistical districts but are included in their original (individual) form. Author's own compilation based on Voigtländer et al. (3).

Figure 1 presents a visualisation of the variables included in the multivariate analysis, which are described in more detail in the following paragraphs.

### Outcome

According to WHO LBW is defined as birth weight lower than 2,500 grams and comprises preterm cases and foetal growth retardation (Supplementary Material 2) (20). LBW increases the risk of neonatal morbidity and mortality and is associated with neurological deficits, developmental delays and chronic diseases later in life (21). Regarding the maternal health impact, mothers of PTB and LBW infants report higher rates of depression, anxiety and impaired mother-infant interaction (22).

### Exposures

#### Small-Area Level

To measure the small-area level resources and stressors, an address-centred approach was chosen (7). First, the participants baseline addresses were checked with the street directory for spelling mistakes. The address of the participant was substituted by information from the first follow up wave if the baseline address was not identifiable in the street directory. This applied to 54 cases from a total of 967 eligible respondents. When the addresses of the participants could not be found in GSV, EKAT or the online portal on environmental noise, we used the nearest available location assuming that the environment resembles the direct living environment at the participant's residence.

For the environmental hazards, the day-evening-night level of long-term average sound level in dB(A) (L_den_) emitted by road traffic, and fine particulate matter of aerodynamic diameter ≤10 μM (PM_10_), emitted by traffic, were retrieved via the online portal on environmental noise and EKAT. EKAT allowed PM_10_ estimates for the requested address. The derived estimates were categorised into four groups (0 to 100 kg/km^2^, >100 to 330 kg/km^2^, <330 to 1.800 kg/km^2^, and higher than 1.800 kg/km^2^). The online portal on environmental noise provided a map with colour coding for five categories of noise pollution. Addresses that had an average L_den_ lower than 55 dB(A) were not represented in the noise maps and were considered as reference group. Accordingly, five categories were derived: ≤55 dB(A), >55 to 60 dB(A), >60 to 65 dB(A), >65 to 70 dB(A), and higher than 70 dB(A) (Supplementary Material 2).

To describe aesthetics characteristics of the built environment, a virtual audit in GSV was designed, based on previously published virtual audit tools (23, 24) and adapted to the subject matter examined here and practical considerations. The list of items has been designed to be short and precise enough to be feasible and not to require any training of the observer (Supplementary Material 3). A 360° assessment of the built environment in GSV was realised in May 2018 following the criteria: Green areas were operationalised as a private garden, a forest or another accumulation of at least ten trees, a green buffer along the road or the pavement (e.g., in the form of grass, trees or hedges) and/or a park/public green. To be rated as a green area at least two of these characteristics needed to be present. Buildings were defined as residential or industrial edifices; garages or junction boxes were not considered in the rating. The condition of the buildings was rated as well-kept if the external façade (including the paint) was not damaged or soiled, no windows were broken, and the building did not seem abandoned. If one of these conditions was violated, it was rated as poor. The street was classified to be in a well-kept condition if at most one hole, crumbling, sizable cracks or uneven section was present, and the road markers were clearly detectable and not damaged. The three items describing the presence of green areas and the condition of the buildings and streets were merged into an index coded as low, middle and high aesthetic (Supplementary Material 2).

The city of Bielefeld is partitioned into 82 statistical districts with areas ranging from 0.21 to 12.4 km^2^ and inhabitants ranging from ~1,200 to 11,500 inhabitants. Within this study, the statistical districts were represented by <5 to 54 participants. The social structure of the small-area level was operationalised following the example of other studies describing the small-area level deprivation (25–27) with the available indicators: proportion of migrants, population density per km^2^, old-age dependency ratio (28), unemployment rate (29), rate on employable people entitled to benefits (ELB-quota) (30); provided by the civil registry of the city of Bielefeld and the statistics of the federal employment agency. For each of the indicators a three-year average (2014 to 2016) was calculated in order to obtain more robust estimates. For all indicators, a high value was considered to be disadvantageous. By z-standardising and adding up the indicators, one summarising index with three levels of deprivation (least to most deprived) was computed (Supplementary Material 2) [following Maier et al. (31)].

#### Individual Level

Individual characteristics have been included to adjust for personal resources (average net household income, migration background, gestational age in weeks, maternal age at birth in years, primiparity, BMI, high blood pressure) and health behaviour (proxy: smoking during pregnancy), (Supplementary Material 2).

### Ethics

The BaBi study obtained approval from the ethical committee of the Medical Faculty of Muenster University as well as the Data Protection Board of Bielefeld University. The project was funded by the Federal Ministry of Education and Research from 2012 to 2017 (grant number: 01ER1202) (16).

### Statistical Analysis

Descriptive statistics were performed to present the sociodemographic characteristics of the BaBi sample and the small-area level as well as the incidence of LBW.

A linear multilevel analysis is performed to investigate the multivariate association of independent variables and birth weight as the dependent variable. The multilevel analysis represents a specific form of regression analysis which allows to examine context and individual factors simultaneously by applying a hierarchical system of regression equations (32). Recognising the dependence of groups or contexts, it takes advantage of clustered data. Moreover, it allows an isolated investigation of small-area and individual level effects adjusted for each other (33, 34).

The multilevel linear regression model will be specified by two-step forward inclusion of the individual level, control variables (Model 1) and the small-area level variables (Model 2) (32, 35). The results will be reported as regression coefficients with 95% confidence intervals (95% CI).

Then, the distributional method for the dichotomisation of continuous outcomes is applied to the results of the multilevel analysis to estimate the marginal differences in proportions with 95% CI for the outcome LBW. The distributional method allows comparing the proportions of a dichotomised continuous outcome, i.e. LBW, with an equal precision as the comparison of means thus avoiding the loss of power associated with dichotomisation (36). A logistic regression model would require a larger sample size and might lead to problems with model convergence (due to demanding model estimation procedures) (37). The distributional method employs the delta method to obtain proportions of the population (defined by a categorical variable) under a certain threshold value by applying estimated normal distribution parameters of the data. The method provides estimates for the difference in proportions for two groups (38). It can produce unadjusted and adjusted comparisons of means and is applicable in linear multilevel regression models with complex data structures.

Consistent with the linear multilevel analysis, estimates for the comparison of proportions for individual and small-area level characteristics are calculated. The assumptions of the multilevel analysis and distributional method were checked with the help of Q-Q-plots, tolerance, Variance Inflation Factors (VIF) and scatterplots (Supplementary Material 4).

After the exclusion of one outlier and 159 (17.83%) cases with missing values, the total sample for the multilevel regression is comprised of *n* = 733. The data analyses were performed with Stata 15 and the user written command reg_distdicho from the Stata module DistDicho (39). All significance tests were performed two-sided and a significance level of α = 0.05 was applied. As the distributional method is based on the estimates of the linear regression model, the p-values are consistent and were only shown for the linear regression model.

## Results

### Sample Distribution

The sample characteristics are shown in Table 1. The mean birth weight is 3,445 g, with an SD of 473 g. 27 newborns weighed less than 2,500 g, which resulted in an LBW incidence of 3%. The newborns are born on average between the 39th and 40th week of gestation. Overall the gestational period varies by 1.14 weeks. 38.6% of all women were primiparous.

**Table 1:** Individual and small-area characteristics (baseline survey 2013 to 2016 and perinatal data, *N* = 892).

	**Sample distribution**				
	***N***	**Mean**	**SD**	**Valid %**	**Missing data *n* (%)**
**OUTCOME**					
Birth weight	892	3,444.71	473.15		0 (0)
Low birth weight	27			3.03	0 (0)
**INDIVIDUAL LEVEL CHARACTERISTICS**					
**Gestational age**	890	39.61	1.14		2 (0.22)
**Primiparity**	342			38.59	0 (0)
**Maternal age at birth**					
18 to 24 years	72			8.07	0 (0)
25 to 29 years	222			24.89	
30 to 34 years	366			41.03	
35 to 49 years	232			26.01	
**Monthly net household income**					
> 4,000€	192			23.91	89 (9.98)
> 2,750€ to 4,000€	262			32.63	
> 1,750€ to 2,750€	188			23.41	
> 800€ to 1,750€	84			10.46	
≤800€	77			9.59	
**Migration background**	326			36.55	0 (0)
**Maternal BMI**					
Underweight (<18.5)	33			3.70	1 (0.11)
Normal weight (18.5 to <25)	570			63.97	
Overweight (25 to <30)	182			20.43	
Obese (≥30)	106			11.90	
**High blood pressure** (self-reported)	53			5.94	0 (0)
**Smoking during pregnancy**	127			15.32	63 (7.06)
**SMALL*****-*** **AREA CHARACTERISTICS**					
**Class variable**					
Statistical districts	892				0 (0)
**L**_**den**_ **due to road traffic [in db(A)]**					
>70 db(A)	69			7.74	1 (0.11)
>65 to 70 db(A)	126			14.14	
>60 to 65 db(A)	114			12.79	
>55 to 60 db(A)	132			14.81	
≤55 db(A)	450			50.51	
**PM**_**10**_ **due to traffic (in kg/km**^**2**^**)**					
≤100 kg/km^2^	50			5.61	0 (0)
>100 to 330 kg/km^2^	182			20.40	
>330 to 1.800 kg/km^2^	562			63.00	
>1.800 kg/km^2^	98			10.99	
**Index on the aesthetic of the built environment**					
High	94			10.54	0 (0)
Middle	557			62.44	
Low	241			27.02	
**Perceived high risk of criminality during daytime**	82			9.03	10 (1.12)
**Social structure**					
Deprivation index					
Highly deprived	301			34.17	11 (1.23)
Middle	284			32.24	
	296			33,60	
Least deprived	301			34.17	

*SD, standard deviation*.

*Data source: BaBi study, EKAT, Online portal on environmental noise in NRW, Google Street View, civil register, employment statistics, statistics on basic security benefits for jobseekers*.

### Multilevel Analysis

Testing the assumptions of the multilevel analysis and distributional method did not indicate any problems with multicollinearity, level one residuals were normally distributed (Supplementary Material 4). The results of the multivariate analysis are presented in Table 2. In brief, the effect of the selected individual and small-area characteristics is small except for the pregnancy-related factors gestational age and primiparity. Besides gestational age, primiparity and the monthly net household income, only the perceived high risk of criminality during the day shows a significant decrease in birth weight.

**Table 2:** Multivariate analysis of (low) birth weight for individual and small-area level characteristics (*n* = 733).

	**Linear regression outcome: birth weight**						**Distributional method outcome: low birth weight**			
	**Model 1**			**Model 2**			**Model 1**		**Model 2**	
	**ß**	**95% CI**	***p***	**ß**	**95% CI**	***p***	**MD**	**95% CI**	**MD**	**95% CI**
**INDIVIDUAL LEVEL CHARACTERISTICS**										
**Gestational age**	193.3	(163.8; 222.8)	0.00^**^	193.3	(163.3; 223.2)	0.00^**^	**–**		**–**	
**Primiparity**	−102.2	(−167.4; −37.0)	0.00^**^	−100.3	(−166.8; −33.8)	0.00^**^	0.009	(0.004; 0.013)^**^	0.008	(0.003; 0.013)^**^
**Maternal age at birth**										
18 to 24 years	−37.4	(−165.9; 91.2)	0.57	−38.7	(−168.6; 91.2)	0.57	0.003	(−0.007; 0.013)	0.003	(−0.007; 0.013)
25 to 29 years (ref.)										
30 to 34 years	−26.8	(−104.5; 50.9)	0.50	−18.6	(−97.1; 59.9)	0.64	0.002	(−0.004; 0.008)	0.001	(−0.004; 0.007)
35 to 49 years	−30.4	(−119.3; 58.6)	0.50	−24.5	(−114.5; 65.5)	0.59	0.002	(−0.004; 0.009)	0.002	(−0.004; 0.008)
**Monthly net household income**										
> 4.000€ (ref.)										
> 2,750€ to 4,000€	−7.1	(−88.4; 74.3)	0.87	−9.0	(−91.5; 73.5)	0.83	0.000	(−0.005; 0.006)	0.001	(−0.005; 0.006)
> 1,750€ to 2,750€	−18.4	(−112.3; 75.4)	0.70	−8.26	(−103.2; 86.7)	0.87	0.001	(−0.005; 0.007)	0.001	(−0.005; 0.007)
> 800€ to 1,750€	−120.4	(−241.2; 0.4)	0.05^*^	−112.8	(−236.4; 11.0)	0.07	0.011	(−0.001; 0.023)	0.010	(−0.002; 0.022)
≤800€	−141.5	(−269.3; −13.8)	0.03^*^	−138.6	(−268.3; −8.8)	0.04^*^	0.014	(0.000; 0.028)^*^	0.013	(−0.001; 0.027)
**Migration background**	11.7	(−54.5; 78.0)	0.73	16.1	(−51.5; 83.8)	0.64	−0.001	(−0.005; 0.003)	−0.001	(−0.005; 0.003)
**BMI**										
Underweight	−76.6	(−228.6; 75.3)	0.32	−85.2	(−238.7; 68.2)	0.28	0.008	(−0.011; 0.028)	0.009	(−0.011; 0.029)
Normal weight (ref.)										
Overweight	67.0	(−11.8; 145.8)	0.10	67.8	(−12.0; 147.6)	0.10	−0.005	(−0.010; 0.000)	−0.005	(−0.010; 0.000)
Obese	104.4	(7.9; 200.8)	0.03^*^	105.5	(7.0; 204.0)	0.04^*^	−0.008	(−0.012; −0.002)^*^	−0.007	(−0.012; −0.002)^*^
**High blood pressure**	−88.6	(−213.3; 36.1)	0.16	−101.5	(−228.3; 25.2)	0.12	0.009	(−0.003; 0.021)	0.010	(−0.003; 0.023)
**Cigarette consumption during pregnancy**	−36.6	(−120.8; 47.7)	0.40	−38.1	(−123.9; 47.8)	0.39	0.003	(−0.003; 0.009)	0.003	(−0.003; 0.009)
**SMALL-AREA LEVEL CHARACTERISTICS**										
**L**_**den**_ **due to road traffic [in db(A)]**										
≤55 db(A) (ref.)										
>55 to 60 db(A)				−0.6	(−93.7; 92.5)	0.99			0.000	(−0.007; 0.007)
>60 to 65 db(A)				30.6	(−68.4; 129.6)	0.55			−0.002	(−0.009; 0.005)
>65 to 70 db(A)				1.0	(−92.4; 94.4)	0.98			−0.000	(−0.007; 0.007)
>70 db(A)				−1.1	(−119.3; 117.1)	0.98			0.000	(−0.009; 0.009)
**PM**_**10**_ **due to traffic (in kg/km**^**2**^**)**										
≤100 kg/km^2^ (ref.)										
>100 to 330 kg/km^2^				−21.3	(−172.8; 130.2)	0.78			0.002	(−0.010; 0.013)
>330 to 1,800 kg/km^2^				13.2	(−129.9; 156.2)	0.86			−0.001	(−0.011; 0.009)
>1,800 kg/km^2^				16.0	(−157.4; 189.5)	0.86			−0.001	(−0.013; 0.011)
**Index on the aesthetic of the built environment**										
High (ref.)										
Middle				−56.5	(−161.0; 48.0)	0.29			0.004	(−0.002; 0.010)
Low				−68.2	(−183.5; 47.1)	0.25			0.005	(−0.003; 0.012)
**Perceived high risk of criminality during daytime**				−114.6	(−222.0; −7.2)	0.04^*^			0.01	(0.000; 0.023)^*^
**Deprivation index**										
Highly deprived				89.1	(−9.7; 187.85)	0.08			−0.007	(−0.013; −0.001)^*^
Middle (ref.)										
Least deprived				43.3	(−53.1; 139.7)	0.38			−0.004	(−0.010; 0.003)

* p < 0.05

** *p < 0.01*.

*CI, confidence interval; MD, marginal differences in proportions*.

*Data source: BaBi study, EKAT, Online portal on environmental noise in NRW, Google Street View, civil register, employment statistics, statistics on basic security benefits for jobseekers*.

#### Linear Regression Model

Of the individual characteristics, the association of gestational age and primiparity with the birth weight is highly significant. With every additional week of the gestational age, the average birth weight increases by almost 200 g ([163.3; 223.2]; *p* < 0.001). The newborns of primiparous women weigh on average 100 g [−166.8; −33.8] less compared to non-primiparas (*p* < 0.001). Additionally, the average birth weight decreased by about 142 g ([−269.3; −13.8]; *p* = 0.030) for the lowest income category, compared to the households with an income of more than 4,000€. In contrast, the birth weight might increase if the BMI of the women is categorised as obese (78.62 [−17.68; 174.91]; *p* = 0.110). Controlling for small-area characteristics does not affect the estimates strongly, and the direction of the association remains stable. The effect sizes remain constant and significant for these variables when adjusting for small-area characteristics.

The other individual characteristics do not show significant associations. However, the results suggest that being older or younger than 25–29 years, having high blood pressure, and smoking during pregnancy tend to decrease the birth weight, while having a migration background marginally increase the birth weight.

The only small-area level characteristic that shows a significant association with birth weight is the perceived criminality: Women that perceive their neighbourhood as unsafe during the day due to criminality, show an about 114 g ([−222.0; −7.2]; *p* = 0.040) lower birth weight of their newborns. Although not significant, the results indicate that environment rated as less aesthetic are associated with a birth weight that is about 70 g lower compared to highly aesthetic built environments (γ_middle = −56.5 [−161.35; 48.0]; γ_low = −68.2 [−183.5; 47.1]).

The daily mean noise pollution due to road traffic, the fine particulate matter due to traffic, and the deprivation index are neither significantly associated with birth weight, nor do they show clear trends according to exposure levels.

#### Distributional Method

Consistent with the linear regression model, the proportion of LBW tends to be significantly higher among primiparous women compared to multiparous women (0.009 [0.004; 0.013]) and among women in the lowest income category (≤800€: 0.013 [−0.001; 0.027]) compared to the highest income category (more than 4,000€ monthly net household income).

Regarding the aesthetic of the built environment, the findings suggest a marginally higher proportion of LBW in built environments rated as being of middle (0.004 [−0.002; 0.010]) or low aesthetic quality (0.005 [−0.003; 0.012]), compared to highly aesthetic areas. The proportion of LBW tends to be significantly higher in neighbourhoods where women fear criminality during daytime compared to those neighbourhoods where women do not (0.010 [0.000; 0.023]). The findings for the environmental hazards, i.e., noise and air pollution, and the deprivation index are inconclusive. They do not indicate a clear direction of the effects and the varying estimates show small to zero effects.

In general, the marginal differences in proportion of LBW are fairly small, ranging from 0 to maximum 1%. The 95% CI are wide which makes the estimation of the slopes unreliable. Additionally, the CI comprise 0, indicating that the effect might go in either direction.

## Discussion

This study analysed the effect of small-area characteristics on LBW, adjusting for individual covariates. Small-area characteristics were assessed using diverse administrative data sources as well as a virtual audit conducted in GSV. These data sources have been linked to data from a birth cohort to realise a multilevel analysis of small-area and individual characteristics on LBW, based on the conceptual model of Voigtländer et al. (3).

The individual characteristics included have proven to be relevant risk factors in prior research decisively influencing the birth weight (40, 41), but in this study only gestational age, primiparity, maternal BMI, and monthly net household income have shown significant effects on LBW. Primiparity, as well as a high maternal BMI, increase the proportion of LBW. With regard to the other individual characteristics, the direction of association indicated in this analysis is consistent with the patterns of associations identified in prior research showing that particularly young women, women with a low monthly net household income or high blood pressure experience a higher risk for LBW (40, 42, 43).

We could not find evidence for consistent negative effects of the environmental hazards or the social structure (operationalised with the deprivation index) on low birth weight. As for the resources and stressors of the small-area context, the results of the multivariate analysis show a trend that newborns of women from less aesthetic and subjectively perceived unsafe neighbourhoods tend to have higher proportions of LBW, which corresponds to our initial hypothesis. Due to the highly heterogenic assessments of the aesthetics of the built environment in the literature, the comparison with the state of research is difficult. Miranda et al. found a significant association between a built environment index on housing damage and LBW and being small for gestational age, after adjusting for various covariates at individual and small-area level (44). For four other indices the estimates attenuated after adjustment (44). With regard to the perceived high risk of criminality during daytime, it should be noted that the effect of criminality is often included in deprivation indices and not investigated separately (13). One study found that criminality at neighbourhood level explained variance in birth weight through economic deprivation: violent crime rates accounted for the majority of the neighbourhood economic disadvantage. Thus, these findings indicate that criminality affects birth weight through different mechanisms (45). In contrast, another analysis identified a significant adverse effect of neighbourhood criminality, independent of small-area economic deprivation (46). Based on these heterogenous findings, we conclude that future research should investigate the mechanisms of perceived criminality on birth outcomes.

Environmental hazards and the social structure of the small-area context do not show clear trends of association with low birth weight in our analysis. The overall impact of noise and air pollution on LBW was non-existing to very low and does not show a consistent direction of the effect, which is mirroring the findings of previous analyses adjusting for maternal and environmental covariates (4, 47). A study conducted in California between 1996 and 2006 on 3,545,177 singleton births, found to have a non-significant increase of LBW for those exposed to PM_2.5_, adjusted for maternal and environmental characteristics (48). In contrast, other studies that only adjusted for maternal covariates identified a statistically significant association of PM_2.5_ and LBW (47). With regard to environmental traffic noise, similar patterns are observed: Only one ecological time series study in Spain found small but significant adverse effects on LBW but did not consider potential confounders (49). Those studies adjusting for maternal and environmental covariates (mostly air pollution) could not find significant effects (4).

As for the deprivation index, our study was only partially able to replicate previous findings. Living in a socioeconomically disadvantaged neighbourhood can have a negative impact on LBW and other birth outcomes according to systematic reviews and meta-analyses. However, most of the studies included adjusted for maternal covariates but not for environmental factors as it was done in this analysis (6, 13). Especially, the measurement of regional deprivation is highly heterogenic in studies examining the effect on health (6, 50, 51). German research showed that the federal system in Germany is one of the key factors impeding homogenous measurement of small-area deprivation on the national level (52). Due to different data structures and data availability at the small-area level, the operationalisation is mostly determined by practical instead of systematic and content-based considerations (50).

This study addresses some of the central challenges identified in prior research. One advantage of the study is the explicit conceptual approach underlying the analysis. By applying the conceptual model of Voigtländer et al. (3), the associations empirically tested derive from theoretical assumptions on the underlying pathways. This allowed to formulate specific a-priori hypotheses and to carefully consider potential confounders and effect modifiers. The linkage of diverse small-area level data sources demonstrates the potential of using routine data of other disciplines for public health research. All data sources fulfilled high quality standards and thus provided a solid basis for the analysis. The objective measures of environmental exposures reduce the risk of recall bias, as they do not rely on participants' perceptions of their neighbourhood (except for the perceived high risk of criminality during daytime). Another strength of the study is the application of the distributional method that allowed to compare the proportions of a dichotomised continuous outcome, i.e., LBW, with the same precision as the comparison of means, thus avoiding loss of power and information (38).

The following limitations have been identified: The restricted sample size of the BaBi study, especially when broken down by the statistical districts of Bielefeld, is one of the main reasons for the imprecise estimates. We have a relatively high number of missing cases (about 17%) in the multivariate analyses. A *post-hoc* power analysis including all variables of the final regression model derived a power of 28.7%. Moreover, the BaBi sample in general is highly educated, and the prevalence of low birth weight in our sample is about half the national average (3 vs. ~6%) (53). Regarding the measurement of small-area characteristics, the address-centred approach might not represent the actual maternal exposure during pregnancy. Additionally, the validity of the virtual audit in GSV is limited due to the sparse coverage of GSV in rural areas and the unstable nature of aesthetic features over time (54). Moreover, the tool has not been tested for its psychometric properties. Since data availability of regional deprivation indicators is challenging, especially on a small-area scale, it determined the selection of indicators, so the domains of education, income and social capital could not be considered. The small-area context is operationalised using administrative units, i.e., the statistical districts. These were the smallest available spatial units for that the statistical office of Bielefeld is providing measures of social composition. It needs to be recognised that the definition of the small-area context might not have any intrinsic meaning for LBW.

## Conclusions

Overall, the empirical evidence linking small-area characteristics and LBW is limited; as well as the explanatory power of the selected small-area characteristics. Our study confirms that obtaining adequate sample size, reliable measure of exposure and using available data for operationalisation of the small-area context represent the core challenges in this field of research. Thus, not only scientific but political efforts are needed to increase the comparability of data structures and data availability in the long term. Improved data availability at the small-area level is not only of interest for health monitoring, but also for social reporting and policy makers in municipalities.

## Data Availability Statement

Study data are available only upon request due to ethical restrictions. Interested researchers may submit requests to Ms. Anja Schmid (Privacy Commissioner, anja.schmid@uni-bielefeld.de). The datasets in Google Street View, EKAT and the online portal on environmental noise in North Rhine-Westphalia analysed are publicly available (https://www.google.de/maps, http://www.ekl.nrw.de/ekat/, http://www.umgebungslaerm.nrw.de).

The registry data that support the findings of this study are held by the Statistical Office of Bielefeld but restrictions apply to the availability of these data, which were used under license for the current study. Data are available from the authors upon reasonable request and with permission of Statistical Office of Bielefeld.

## Ethics Statement

The BaBi study includes human participants and has been reviewed and approved by the ethics committee of the Medical Faculty of Muenster University and the Data Protection Board of Bielefeld University. The participants provided their written consent to participate in the study.

## Author Contributions

LW designed the analysis strategy, conducted the statistical analyses, and drafted the manuscript. OR contributed to the conception and design of the study. JB and OS advised on the statistical analysis. OS, JB, and OR revised the manuscript critically for intellectual content. JS and OR are head of the study. All authors read and approved the final version of the manuscript.

## Conflict of Interest

The authors declare that the research was conducted in the absence of any commercial or financial relationships that could be construed as a potential conflict of interest.

## Abbreviations

BaBi: “Gesundheit von Babys und Kindern in Bielefeld” (Health of infants and children in Bielefeld/Germany)
BMI: body mass index
EKAT: “Online Emissionskataster Luft NRW” (online emission inventory “air”)
ELB-quota: rate of employable people entitled to social benefits
GSV: Google Street View
LBW: low birth weight
L_den_: day-evening-night level of long-term average sound level in dB(A)
PM_10_: particulate matter of aerodynamic diameter ≤10 μM
PTB: preterm birth
WHO: World Health Organization.
