# Supplementary material for: Small-Area Factors and Their Impact on Low Birth Weight—Results of a Birth Cohort Study in Bielefeld, Germany
Source: Front Public Health. 2020 Apr 28;8:136. doi: 10.3389/fpubh.2020.00136 (PMC7199350; doi:10.3389/fpubh.2020.00136)
Supplement: Supplementary file 1 [file Data_Sheet_1.docx]

Supplementary Material 1: statistical selection of dependent variables with bivariate statistics

Table 1 T-tests analysing the bivariate association of binary individual and small-area covariates with birth weight

|  |  | **T-tests for individual and small-area characteristics with birth weight** | | |
| --- | --- | --- | --- | --- |
|  | | **n** | **mean** | **p** |
| **individual characteristics** | **primiparity** |  |  |  |
|  | no | 550 | 3,486.2 | 0.001** |
|  | yes | 342 | 3,378.1 |  |
|  | **risk pregnancy** |  |  |  |
|  | no | 480 | 3,442.5 | 0.880 |
|  | yes | 407 | 3,447.3 |  |
|  | **marital status** |  |  |  |
|  | with partner | 42 | 3,333.6 | 0.114 |
|  | without partner | 847 | 3,451.8 |  |
|  | **migration background** |  |  |  |
|  | no | 566 | 3,454.0 | 0.440 |
|  | yes | 326 | 3,428.6 |  |
|  | **diabetes** |  |  |  |
|  | no | 811 | 3,442.7 | 0.629 |
|  | yes | 79 | 3,469.7 |  |
|  | **high blood pressure** |  |  |  |
|  | no | 839 | 3,452.6 | 0.047* |
|  | yes | 53 | 3,319.4 |  |
|  | **perceived chronic stress** |  |  |  |
|  | no | 571 | 3,459.2 | 0.156 |
|  | yes | 315 | 3,412.0 |  |
|  | **perceived acute stress** |  |  |  |
|  | no | 548 | 3,446.4 | 0.898 |
|  | yes | 336 | 3,442.1 |  |
|  | **smoking during pregnancy** |  |  |  |
|  | no | 702 | 3,458.6 | 0.059* |
|  | yes | 127 | 3,372.9 |  |
|  | **alcohol consumption during pregnancy** |  |  |  |
|  | no | 660 | 3,441.3 | 0.718 |
|  | yes | 232 | 3,454.4 |  |
|  | **physical activity** |  |  |  |
|  | no | 411 | 3,439.4 | 0.781 |
|  | yes | 471 | 3,448.3 |  |
| **small-area characteristics** | **Perceived high risk of criminality during daytime** |  |  |  |
|  | no | 800 | 3,457.3 | 0.012* |
|  | yes | 82 | 3,319.8 |  |
|  | **availability of shopping facilities** |  |  |  |
|  | no | 46 | 3,391.5 | 0.434 |
|  | yes | 844 | 3,447.7 |  |
| * p<0.05; ** p<0.01  Author’s own compilation. *Data source:* BaBi study. | | | | |

Table 2 Spearman’s rank correlation analysing the bivariate association of categorical and continuous variables with birth weight

|  |  | **Spearman’s rank correlation for individual and small-area characteristic with birth weight** | | |
| --- | --- | --- | --- | --- |
|  |  | **n** | **r^a^** | **p** |
| **individual characteristics** | gestational age | 890 | 0.387 | <0.001** |
|  | maternal age | 891 | 0.060 | 0.072 |
|  | highest vocational education | 868 | 0.027 | 0.428 |
|  | occupational status | 881 | 0.018 | 0.584 |
|  | monthly net household income | 803 | 0.103 | 0.003* |
|  | maternal BMI | 891 | 0.078 | 0.020* |
|  | social support | 857 | 0.065 | 0.058 |
|  | internal control expectations | 889 | -0.015 | 0.646 |
|  | external control expectations | 888 | -0.033 | 0.319 |
| **small-area characteristics** | L_den_ due to road traffic (in dB(A)) | 891 | -0.029 | 0.387 |
|  | PM_10_ due to traffic (in kg/km^2^) | 892 | -0.024 | 0.478 |
|  | index on the aesthetic of the built environment | 892 | 0.049 | 0.144 |
|  | deprivation index | 892 | 0.014 | 0.673 |
| * p<0.05; ** p<0.01; ^a^Spearman’s rho rank correlation coefficient  Author’s own compilation. *Data source:* BaBi study, EKAT, Online portal on environmental noise in NRW, Google Street View, civil register, employment statistics, statistics on basic security benefits for jobseekers. | | | | |
