# Supplementary material for: Small-Area Factors and Their Impact on Low Birth Weight—Results of a Birth Cohort Study in Bielefeld, Germany
Source: Front Public Health. 2020 Apr 28;8:136. doi: 10.3389/fpubh.2020.00136 (PMC7199350; doi:10.3389/fpubh.2020.00136)
Supplement: Supplementary file 2 [file Data_Sheet_2.docx]

Supplementary Material 2: measurement of individual and small-area characteristics

Table 3 Measurement of individual and small-area characteristics

| **Variable** | **Specification** | **Data source** |
| --- | --- | --- |
| *outcome* | | |
| low birth weight | birth weight < 2,500 gram (operationalised according to WHO standards) | Perinatal data |
| *Small-area exposures* | | |
| L_den_ | Road traffic has been selected as the noise emitter for the latest available computation in 2017. The highest category that affected the residential building was selected as the estimate for noise pollution.  0 ‘>70 dB(A)’  1 ‘>65 to 70 dB(A)’  2 ‘>60 to 65 dB(A)’  3 ‘>55 to 60 dB(A)’  4 ‘≤55 dB(A) | Online portal on environmental noise |
| PM_10_ | The pollutant PM_10_, the emitter group traffic and the survey year of 2013 were selected  0 ‘>1.800 kg/km^2^’  1 ‘>330 to 1.800 kg/km^2^’  2 ‘>100 to 330 kg/km^2^’  3 ‘≤ 100 kg/km^2^’ | EKAT |
| index on the aesthetics of the built environment | Assessment in the virtual audit in GSV. If the criteria in Table 1 (Additional file 3) were fulfilled, the item was scored with one point, otherwise it was coded as zero. Then a sum score was calculated which was categorised into three groups. Those environments that scored zero or one point were coded as low aesthetic, two points as middle and three as high aesthetic. | GSV |
| perceived high risk of criminality during daytime | Self-reported answer to the question: Do you feel unsafe in your nearest living environment during day?  Coded binary (yes/no) | BaBi study |
| deprivation index | Consists of three-year averages (2014-2016) of the indicators: *proportion of migrants gives, population density per km2, old-age dependency ratio (30), unemployment rate (31), rate on employable people entitled to benefits (ELB-quota) (32)*, a high value was considered to be disadvantageous By z-standardising and adding up the indicators, one summarising index with three levels of deprivation (least to most deprived) was computed (Additional file 2) (following (33)). | Civil registry, statistic of the BA |
| *Individual covariables* | | |
| maternal age | The maternal age at birth was calculated with the help of the birth date of the mother and the newborn. By calculating the time period in years between the two dates, the maternal age was calculated. Then it was categorised into four age categories (18 to 24, 25 to 29, 30 to 34 and 35 to 49). | BaBi study |
| average monthly net income of the household | The average net household income was measured by the concrete amount in € or by intervals ranging from 0 to 20,000€ in steps of 250€, if the participant would refuse the first option. The net household income per month is scaled ordinal with five categories (≤ 800€; > 800€ to 1,750€; > 1,750€ to 2,750€; > 2,750€ to 4,000€; > 4,000€). | BaBi study |
| migration background | The dichotomised variable is derived with the help of the country of birth of the BaBi participant and of both parents as well as the mother tongue. Women were rated to have a migration background, if: they were not born in Germany and have at least one parent that was not born in Germany, or they were born in Germany and both parents were born abroad, or their mother tongue is not German. The specification of having a migration background includes women with a first, second or third generation back-ground. | BaBi study |
| gestational age | The gestational age of the newborn was estimated with the help of the expected birth date and the actual date. By subtracting the actual birth date from the expected one, the difference in weeks of these two dates was calculated. This difference was then subtracted from 40 (the average duration of pregnancy in weeks) which yields the gestational age in weeks (28 – 42) | Perinatal data |
| primiparity | As documented in the perinatal data (yes/no) | Perinatal data |
| Maternal BMI | The BMI has been calculated using baseline data (weight (kg) / [height (m)]^2^ which complies to WHO standards (46, 47)). It was categorised according to the international classification of adult under-/overweight and obesity. A BMI under 18.5 is rated ‘underweight’, 18.5 up to 25 is ‘normal weight’, 25 up to 30 is considered ‘overweight’ and a BMI of 30 and higher is ‘obese’. | BaBi study/ perinatal data |
| high blood pressure | The binary variable included self-reported and by physician diagnosed diseases (yes/no). | BaBi study |
| smoking during the pregnancy | Several survey questions on the cigarette consumption (d1_06 – d1_07_3) have been merged into one item each, to indicate whether the participant smoked during the pregnancy (‘no’ and ‘yes’). The ‘no’ categories include women that never smoked cigarettes, drank alcohol or stopped the consumption before the pregnancy. The women that declared to have consumed cigarettes or alcohol during the first trimester and afterwards were coded as ‘yes’. | BaBi study |

Data sources: *Data source:* BaBi study, EKAT, Online portal on environmental noise in NRW, Google Street View, civil register, employment statistics, statistics on basic security benefits for jobseekers.
