# Supplementary material for: Small-Area Factors and Their Impact on Low Birth Weight—Results of a Birth Cohort Study in Bielefeld, Germany
Source: Front Public Health. 2020 Apr 28;8:136. doi: 10.3389/fpubh.2020.00136 (PMC7199350; doi:10.3389/fpubh.2020.00136)
Supplement: Supplementary file 3 [file Data_Sheet_3.docx]

Supplementary Material 3: Protocol of inclusion criteria for the virtual audit in Google Street View

Table 4 Protocol of inclusion criteria for the virtual audit in Google Street View

| **Item** | **Specification** | **Inclusion criteria** |
| --- | --- | --- |
| Presence of green area | 0 ‘no’  1 ‘yes’ | The area is classified as a “green area” if at least two of the following criteria are fulfilled:   - private garden - forest - other accumulation of at least 10 trees (whereas a tree is defined to be at least as high as the first floor of the surrounding buildings) - buffer among the majority of the road/pavement (e.g. in form of grass, trees, hedges) - parks/public green (including play grounds if grassy areas exist) |
| Condition of the buildings | 0 ‘well-kept’  1 ‘poor condition’ | The buildings’ condition is rated well-kept, if all visible buildings look well maintained meaning:   - the paint looks to be in good condition - the plaster is neither damaged nor soiled (e.g. with graffiti) - the windows are not broken - the building does not seem abandoned |
| Condition of the streets | 0 ‘well-kept’  1 ‘poor condition’ | The streets’ condition is rated to be in a good condition, if   - there are no (or maximum 1) holes, sizable cracks, crumbling or uneven sections and - the road markers are clearly detectable and not damaged (if existent). |
| Author’s own compilation based on (Bethlehem et al., 2014; Chow et al., 2014; Hoehner et al., 2007; Sallis et al., 2015). | | |
