# Supplementary material for: Small-Area Factors and Their Impact on Low Birth Weight—Results of a Birth Cohort Study in Bielefeld, Germany
Source: Front Public Health. 2020 Apr 28;8:136. doi: 10.3389/fpubh.2020.00136 (PMC7199350; doi:10.3389/fpubh.2020.00136)
Supplement: Supplementary file 4 [file Data_Sheet_4.docx]

Supplementary Material 4: Testing the assumptions of the multilevel analysis and distributional method

# Assumption of normal distribution

Table 5a Statistics on the distributional parameters (n=734)

|  | **min** | **max** | **mean** | **SD** | **skewness** | **curtosis** |
| --- | --- | --- | --- | --- | --- | --- |
| birth weight (in g) | 970 | 4,830 | 3,433.34 | 476.13 | -0.13 | 0.74 |
| gestational age | 28 | 42 | 39.60 | 1.12 | -2.51 | 16.59 |
| standardised residual | -2.50 | 3.01 | 0.00 | 0.99 | 0.24 | -0.17 |

Table 5b Statistics on the distributional parameters after excluding one outlier (n=733)

|  | **min** | **max** | **mean** | **SD** | **skewness** | **curtosis** |
| --- | --- | --- | --- | --- | --- | --- |
| birth weight (in g) | 2,080 | 4,830 | 3,436.70 | 467.66 | 0.05 | -0.04 |
| gestational age | 35 | 42 | 39.61 | -1.30 | -1.29 | 2.33 |
| standardised residual | -2.50 | 3.01 | 0.00 | 0.04 | 0.24 | -0.18 |


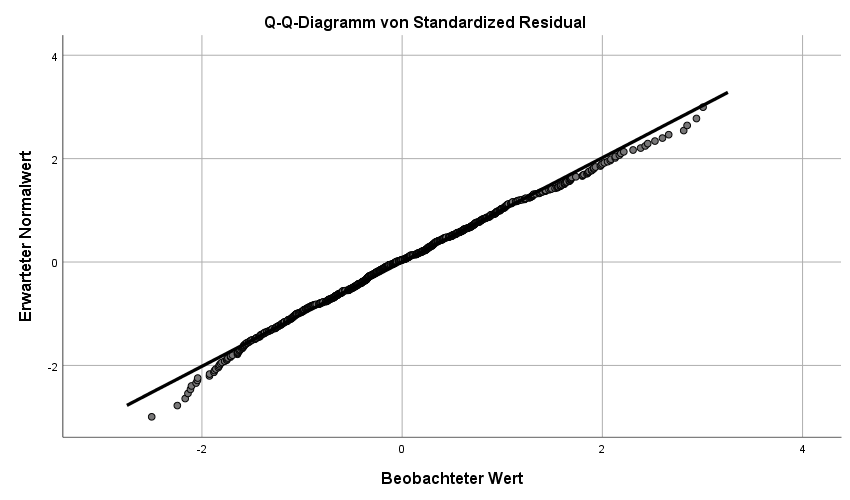


Expected normal value

Observed value

Figure 1 Q-Q-Plot of the standardised residual (n=734)

Author’s own compilation. *Data source:* BaBi study.


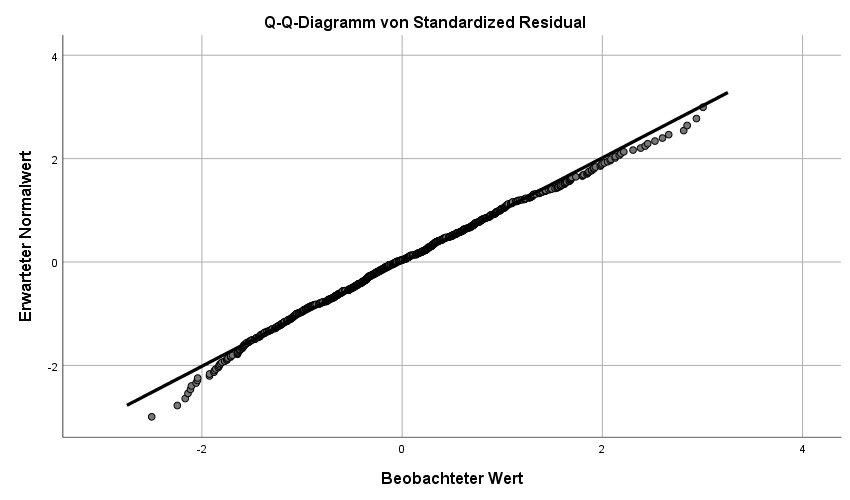


Observed value

Expected normal value

Figure 2 Q-Q-Plot of the standardised residual after excluding one outlier (n=733)

Author’s own compilation. *Data source:* BaBi study.

# Assumption of multicollinearity

Table 6a Analysis of multicollinearity (n=734)

|  | **analysis of collinearity** | |
| --- | --- | --- |
|  | **tolerance** | **VIF** |
| gestational age | 0.981 | 1.019 |
| primiparity | 0.902 | 1.108 |
| maternal age at birth | 0.796 | 1.256 |
| monthly net household income | 0.766 | 1.305 |
| migration background | 0.890 | 1.124 |
| Body Mass Index | 0.962 | 1.039 |
| high blood pressure | 0.947 | 1.056 |
| smoking during pregnancy | 0.912 | 1.096 |
| daily mean of noise pollution due to road traffic | 0.932 | 1.073 |
| fine particulate matter due to traffic | 0.928 | 1.078 |
| index on the aesthetic of the built environment | 0.960 | 1.042 |
| Perceived high risk of criminality during daytime | 0.964 | 1.037 |
| deprivation index | 0.874 | 1.144 |
| Author’s own compilation. *Data source:* BaBi study. | | |

Table 6b Analysis of multicollinearity after exclusion of an outlier (n=733)

|  | **analysis of collinearity** | |
| --- | --- | --- |
|  | **tolerance** | **VIF** |
| gestational age | 0.966 | 1.035 |
| primiparity | 0.902 | 1.109 |
| maternal age at birth | 0.795 | 1.258 |
| monthly net household income | 0.766 | 1.306 |
| migration background | 0.889 | 1.124 |
| Body Mass Index | 0.926 | 1.079 |
| high blood pressure | 0.944 | 1.060 |
| smoking during pregnancy | 0.912 | 1.096 |
| daily mean of noise pollution due to road traffic | 0.932 | 1.073 |
| fine particulate matter due to traffic | 0.923 | 1.084 |
| index on the aesthetic of the built environment | 0.958 | 1.043 |
| Perceived high risk of criminality during daytime | 0.963 | 1.038 |
| deprivation index | 0.875 | 1.143 |
| Author’s own compilation. *Data source:* BaBi study. | | |

# Assumptions of homoscedasticity and linearity and the identification of outliers


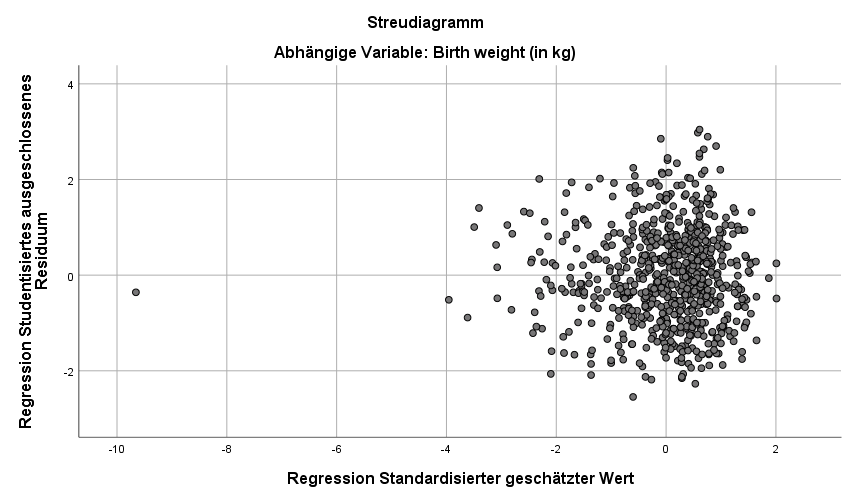


Studentised excluded residual

Standardised value

Figure 3 Scatterplot of the standardised residual for the outcome birth weight (n=734)

Author’s own compilation. *Data source:* BaBi study.


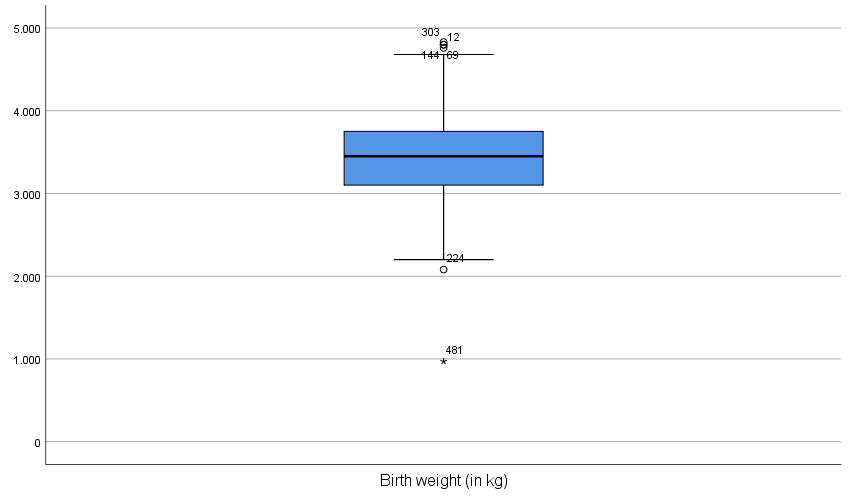


Birth weight (in g)

Figure 4 Boxplot for the outcome variable birth weight (n=734)

Author’s own compilation. *Data source:* BaBi study.


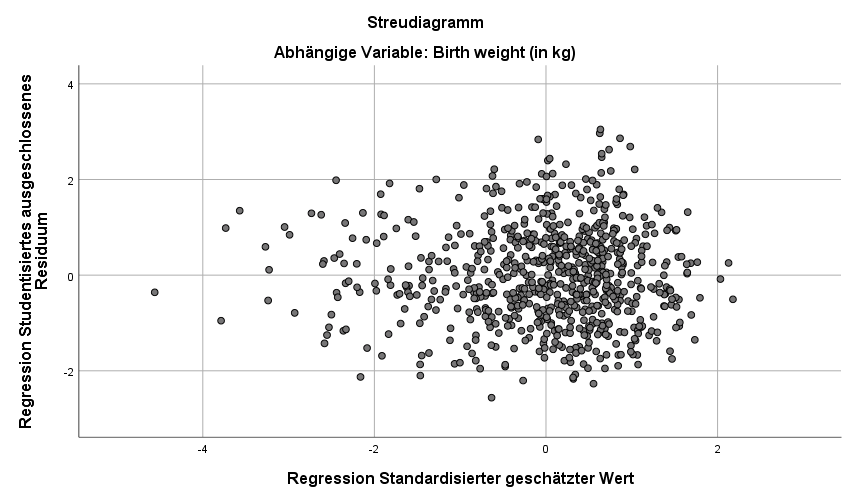


Standardised value

Studentised excluded residual

Figure 5 Scatterplot of the standardised residual for the outcome birth weight after excluding one outlier (n=733)

Author’s own compilation. Data source: BaBi study.
